# Supplementary material for: Morton’s Neuroma or Intermetatarsal Bursitis—A Prospective Diagnostic Study of Intermetatarsal Pain
Source: Diagnostics (Basel). 2025 May 26;15(11):1339. doi: 10.3390/diagnostics15111339 (PMC12154574; doi:10.3390/diagnostics15111339)
Supplement: Supplementary file 1 [file diagnostics-15-01339-s001.zip › diagnostics-3604088-supplementary.pdf]

Supplementary materials

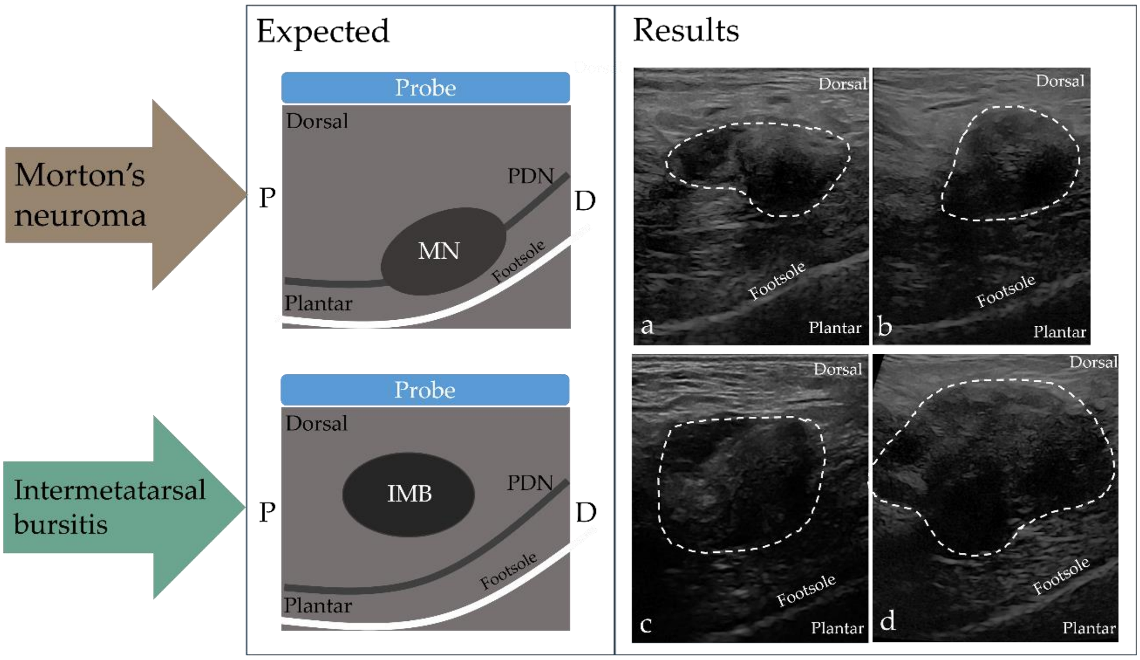

Figure S1. Schematic diagram of the expected method of differentiation based on our predefined ultrasound (US) criteria correlated to our US results. US scans from patients with MN on MRI (a, b) could not be distinguished from US scans from patients with IMB on MRI (c, d). P = proximal, D = distal, PDN = plantar digital nerve.

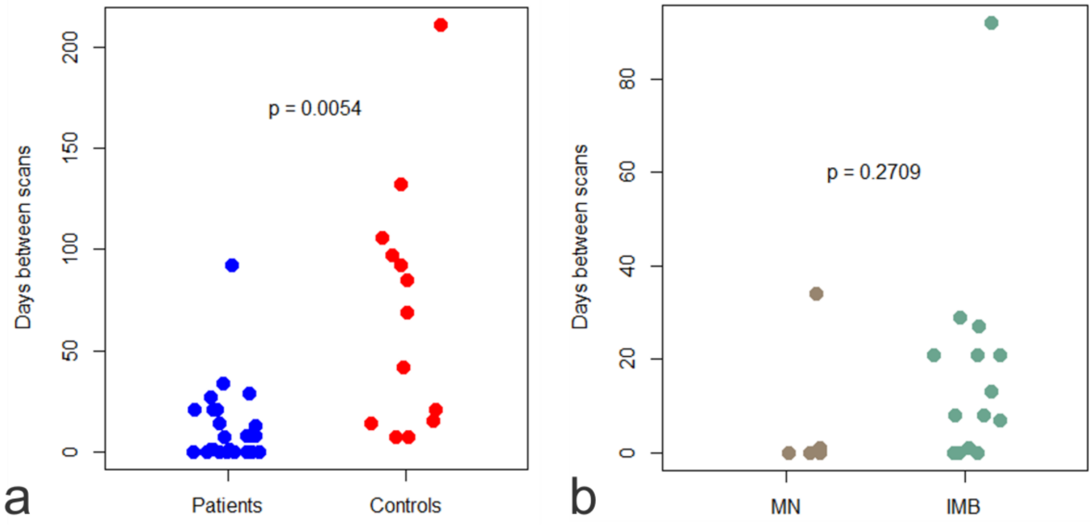

Figure S2. Overview of the time between the ultrasound and the MRI scan for the patient and controls (a) and the patients sub grouped by diagnosis (b).

Table S1. Differences in symptoms between IMB and MN.
BH = Benjamini-Hochberg correction for multiple comparisons.

Symptom	p_value	p_value_BH
Shooting	0.1280	0.6756
Sharp	0.3378	0.6756
Pressing	0.2565	0.6756
Sensation	0.2565	0.6756
Dull	0.4678	0.7485
Burning	0.5696	0.7594
Stabbing	1.0000	1.0000
Rock in shoe	1.0000	1.0000
Aggravating		
Narrow shoes	0.0379	0.1138
Walking	0.5304	0.7957
Hard surface	1.0000	1.0000
Alleviating		
Rest	0.1280	0.6398
Feet up	0.3047	0.7617
Shoes off	0.5696	0.9493
Massage foot	1.0000	1.0000
Shoes	1.0000	1.0000

Table S2. STROBE checklist.

	Item No	Recommendation
**Title and abstract**	1	(*a*) Indicate the study's design with a commonly used term in the title or the abstract<br>*Morton's Neuroma or Intermetatarsal Bursitis – **Prospective Diagnostic Study** of Intermetatarsal Pain*
		(*b*) Provide in the abstract an informative and balanced summary of what was done and what was found<br>*Abstract line 17-24, page 1*
**Introduction**		
Background/rationale	2	Explain the scientific background and rationale for the investigation being reported<br>*Introduction line 45-60, page 2*
Objectives	3	State specific objectives, including any prespecified hypotheses<br>*Introduction line 60-64, page 2*

**Methods**		
Study design	4	Present key elements of study design early in the paper<br>*Methods line 71-72, page 2*
Setting	5	Describe the setting, locations, and relevant dates, including periods of recruitment, exposure, follow-up, and data collection<br>*Methods line 73-74 and 81-88, page 2*
Participants	6	(*a*) *Case-control study*—Give the eligibility criteria, and the sources and methods of case ascertainment and control selection. Give the rationale for the choice of cases and controls<br>*Methods line 84-89, page 2 and line 90-103, page 2-3*
		Case-control study—For matched studies, give matching criteria and the number of controls per case<br>*Not applicable*
Variables	7	Clearly define all outcomes, exposures, predictors, potential confounders, and effect modifiers. Give diagnostic criteria, if applicable<br>*Methods line 137-140 (primary) and line 98-103 (secondary). US diagnostic criteria reported in line 117-122, page 3 and MRI diagnostic criteria in line 131-136, page 4 supported by figure 2, page 4*
Data sources/ measurement	8*	For each variable of interest, give sources of data and details of methods of assessment (measurement). Describe comparability of assessment methods if there is more than one group<br>*Described in method line 108-117, page 3 (US) and line 123-131, page 3-4*
Bias	9	Describe any efforts to address potential sources of bias<br>*Methods line 140-143, page 4*
Study size	10	Explain how the study size was arrived at<br>*See Figure 1, page 3.*
Quantitative variables	11	Explain how quantitative variables were handled in the analyses. If applicable, describe which groupings were chosen and why<br>*Methods line 145-146, page 4*
Statistical methods	12	(*a*) Describe all statistical methods, including those used to control for confounding<br>*Not applicable*
		(*b*) Describe any methods used to examine subgroups and interactions<br>*Methods line 146-148, page 4*
		(*c*) Explain how missing data were addressed<br>*Methods line 148-149, page 4*
		(*d*) *Case-control study*—If applicable, explain how matching of cases and controls was addressed<br>*Not applicable*
		(*e*) Describe any sensitivity analyses

Not relevant

Continued on next page

**Results**		
Partici-pants	13*	(a) Report numbers of individuals at each stage of study—eg numbers potentially eligible, examined for eligibility, confirmed eligible, included in the study, completing follow-up, and analysed<br>*Methods line 173-179, page 5*
		(b) Give reasons for non-participation at each stage<br>*See Figure 1, page 3.*
		(c) Consider use of a flow diagram<br>*See flowchart in Figure 1, page 3.*
Descrip-tive data	14*	(a) Give characteristics of study participants (eg demographic, clinical, social) and information on exposures and potential confounders<br>*See Table 1, page 5.*
		(b) Indicate number of participants with missing data for each variable of interest<br>*See Table 1, page 5*
Outcome data	15*	*Case-control study*—Report numbers in each exposure category, or summary measures of exposure<br>*See Table 2, page 6*
Main re-sults	16	(*a*) Give unadjusted estimates and, if applicable, confounder-adjusted estimates and their precision (eg, 95% confidence interval). Make clear which confounders were adjusted for and why they were included<br>*See Table 2, page 6*
		(*b*) Report category boundaries when continuous variables were categorized<br>*Not applicable*
		(*c*) If relevant, consider translating estimates of relative risk into absolute risk for a meaningful time period<br>*Not relevant*
Other analyses	17	Report other analyses done—eg analyses of subgroups and interactions, and sensitivity analyses<br>*Results line 258-274, page 9*
**Discussion**		
Key re-sults	18	Summarise key results with reference to study objectives<br>*Discussion line 285-293, page 10-11*
Limita-tions	19	Discuss limitations of the study, taking into account sources of potential bias or imprecision. Discuss both direction and magnitude of any potential bias<br>*Discussion line 374-396, page 12-13*
Interpre-tation	20	Give a cautious overall interpretation of results considering objectives, limitations, multiplicity of analyses, results from similar studies, and other relevant evidence
Generali-sability	21	Discuss the generalisability (external validity) of the study results

		Item no. 20+21 is addressed under each result interpretation, lines 304-373, page 11-12
**Other information**		
Funding	22	Give the source of funding and the role of the funders for the present study and, if applicable, for the original study on which the present article is based<br>*Line 417-418, page 13*

*Give information separately for cases and controls in case-control studies and, if applicable, for exposed and unexposed groups in cohort and cross-sectional studies.

Note: An Explanation and Elaboration article discusses each checklist item and gives methodological background and published examples of transparent reporting. The STROBE checklist is best used in conjunction with this article (freely available on the Web sites of PLoS Medicine at http://www.plosmedicine.org/, Annals of Internal Medicine at http://www.annals.org/, and Epidemiology at http://www.epidem.com/). Information on the STROBE Initiative is available at www.strobe-statement.org.
